# Parametric investigation of W-EDM factors for machining AM60B conductive biomaterial

**DOI:** 10.1038/s41598-023-50777-y

**Published:** 2024-01-02

**Authors:** M. Diviya, J. Jebin Joel, M. Subramanian, T. Balasubramanian, A. V. Madhusuthan, N. Monish, Nasim Hasan

**Affiliations:** 1 Department of Computer Science and Engineering, Amrita School of Computing, Amrita Vishwa Vidyapeetham - Chennai Campus, Chennai, Tamil Nadu 601103 India; 2 Department of Mechanical Engineering, St. Joseph’s College of Engineering, Old Mamallapuram Road, Chennai, Tamil Nadu 600119 India; 3 Department of Mechanical Engineering, College of Engineering and Technology, Mettu University, Mettu, Oromia Ethiopia

**Keywords:** Engineering, Materials science

## Abstract

Wire—electrical discharge machining (W-EDM) is a precise and efficient non-traditional technology employed to cut intricate shapes in conductive biomaterials. These biomaterials are challenging to machine using traditional methods. This present study delves into the impact of various process parameters, namely discharge duration (D_dur_), spark gap time (S_time_), discharge voltage (D_volt_), and wire advance rate rate (W_adv_). This research evaluates the impact of several factors on response variables, namely the machining rate (MR) and surface irregularity (SR), during the machining process of the AM60B magnesium alloy. The confirmation of the material used in the machining process is achieved via the utilisation of a scanning electron microscopy (SEM) image in conjunction with an energy dispersive spectroscopic (EDS) image. The experiment is designed as L9 orthogonal array by using Taguchi's approach, taking into account 4 factors with 3 levels. The objective of this experiment is to ascertain the most favourable values for machining parameters while working with AM60B magnesium alloy using brass wire. Through analysis of variance (ANOVA), the study confirms that wire advance rate (43.10%) is the most influencing parameter for machining rate and surface irregularity followed by spark gap time (33.91%) and discharge duration (11.48%). Additionally, The TOPSIS-CRITIC and the desirability approach were used in order to determine the optimum parameter combinations that provide the most favourable combined output. Confirmatory testing is used to evaluate the efficiency of the stated ideal conditions. The maximum improvement in Desirability approach is obtained at 4.56% and 4.193% for MR and SR respectively. The maximum improvement in TOPSIS approach is obtained at 1.77% and 2.78% for MR and SR respectively.

## Introduction

With the upsurge on environmental concerns, the development of biodegradable biomaterials aligns with the goals of sustainability and reduced ecological impact^1,2^. The biocompatible magnesium alloys hold substantial promise across industries, especially in healthcare as these alloys have the potential to transform medical practices, enhance patient outcomes, and will contribute to a more sustainable and innovative future^3^. The pursuit of intricate implant shapes drives innovation in materials science, engineering and medical research. Magnesium alloys can be challenging to machine using traditional machining processes as a result of limited thermal conductivity, high reactivity, low modulus of eleasticity and high ductility which causes low machinability of AM60B magnesium alloys with surface irregularities and less accuracy^4^. Wire electrical discharge machining (WEDM) enables the manufacturing of patient specific implants that were previously difficult using traditional machining methods^5^. Wire Electrical Discharge Machining (WEDM) is a favoured specialised machining procedure because to its precision and adaptability. Because of its remarkable accuracy in forming intricate shapes, it is essential in industries needing delicate components^6–8^. Hard materials are challenging for traditional methods to cut through, such as unusual alloys and hardened steels which is easily achieved through wire-EDM. Its minimal heat generation and non-contact characteristics make it ideal for handling delicate or heat-sensitive objects. When there is no tool wear in the wire electrode, the consistency of machined products is preserved and tool life is increased^9^. Additionally, production is made user-friendly and productive by its automation characteristics. WEDM is particularly suited for applications where reducing heat-affected zones, minimising burrs, and achieving exact tolerances are crucial. Even though it might not be the fastest method, its unique qualities make it a crucial tool in precision machining and difficult item creation^10^. One significant limitation of traditional machining methods is the deterioration of surface qualities due to the formation of burrs and cutting tool built-up during machining of magnesium alloys^11^. Additionally, the high reactivity of magnesium alloys can lead to the risk of ignition at elevated cutting speeds during the machining process^12,13^. WEDM represents one amongst the many advanced unconventional machining technologies for producing complicated forms and features with great precision utilising conducting material. WEDM is one of the best alternatives making it a suitable choice for producing high quality implants with minimal risk to patient health and safety^14^. The researchers have studied the WEDM method for a variety of conductive metals and composites^15,16^, but relatively few studies have been performed studies examining its impact during WEDM process upon the efficacy on Mg-based alloys and composites. As a result, this research focuses on the machining of AM60B magnesium alloy utilising the WEDM technique. AM60B is a kind of magnesium alloy that has a relatively low density, which renders it extremely light-weight, as well as outstanding castability, ductility, and thermal conductivity^17,18^. Furthermore, establishing an appropriate parameter setting in WEDM is a key topic for lowering machining costs.

Seshadhri et al. conducted a study to examine the impact of process parameters in WEDM on the material removal rate (MRR) and surface roughness (SR) of a magnesium alloy AZ31 matrix that had been reinforced with a mixture of seashell powder (2 wt%) and zirconium dioxide (10 wt%). The ANOVA findings showed that pulse current had a statistically significant influence on both Material Removal Rate (MRR) and Surface Roughness (Ra).^19^. Mandal et al., outlined WEDM as a prominent non-conventional manufacturing process. Taguchi was used in the experiments. and TOPSIS built around entropy was used to combine several response settings to one central factor. ANOVA was utilized to validate the optimal outcomes^20^. Dewangan et al., utilized A flexible TOPSIS-based technique for multiple-criterion selection to optimise variables in the EDM process. The aforementioned attributes include pulse current, pulse-on duration, tool operation duration, and tool lifespan. The optimisation process was conducted with consideration given to several surface integrity criteria, including the White Layer Thickness (WLT), surface fracture density, and surface roughness^21^. Bikas et al., aimed to optimize WEDM parameters using a hybrid optimization technique, the desirability based TOPSIS for parameters like kerf width and surface roughness^22^. Singh et al., in his research work utilized TOPSIS-CRITIC (criteria importance through criteria inter-correlation) technique to determine the effectiveness of optimization of multiple response green wire electrical discharge variables for machining on H21 steel^23^. In addition to several attributes like pulse on time, pulse off time, wire tension and wire feed, peak current appeared to have the greatest influence on surface roughness^24,25^.

The analysis of the literature found that there are few research papers on WEDM of magnesium alloys and a notable lack of research on AM60B magnesium alloy machining. Machining alters the surface characteristics of the biocompatible AM60B, affecting its degradation. As a result, the machining properties of the AM60B magnesium alloy must be investigated. Furthermore, it has been stated in the literature that TOPSIS has been extremely effective in WEDM modelling; consequently, we want to apply this approach. As a result, the current study assessed and optimised WEDM process parameters such as discharge duration, spark gap time, for machining frequency and discharging voltage surface irregularity of AM60B Mg-alloy using the TOPSIS-CRITIC method and the desirability approach.

## Materials and methods

**Figure 1:**
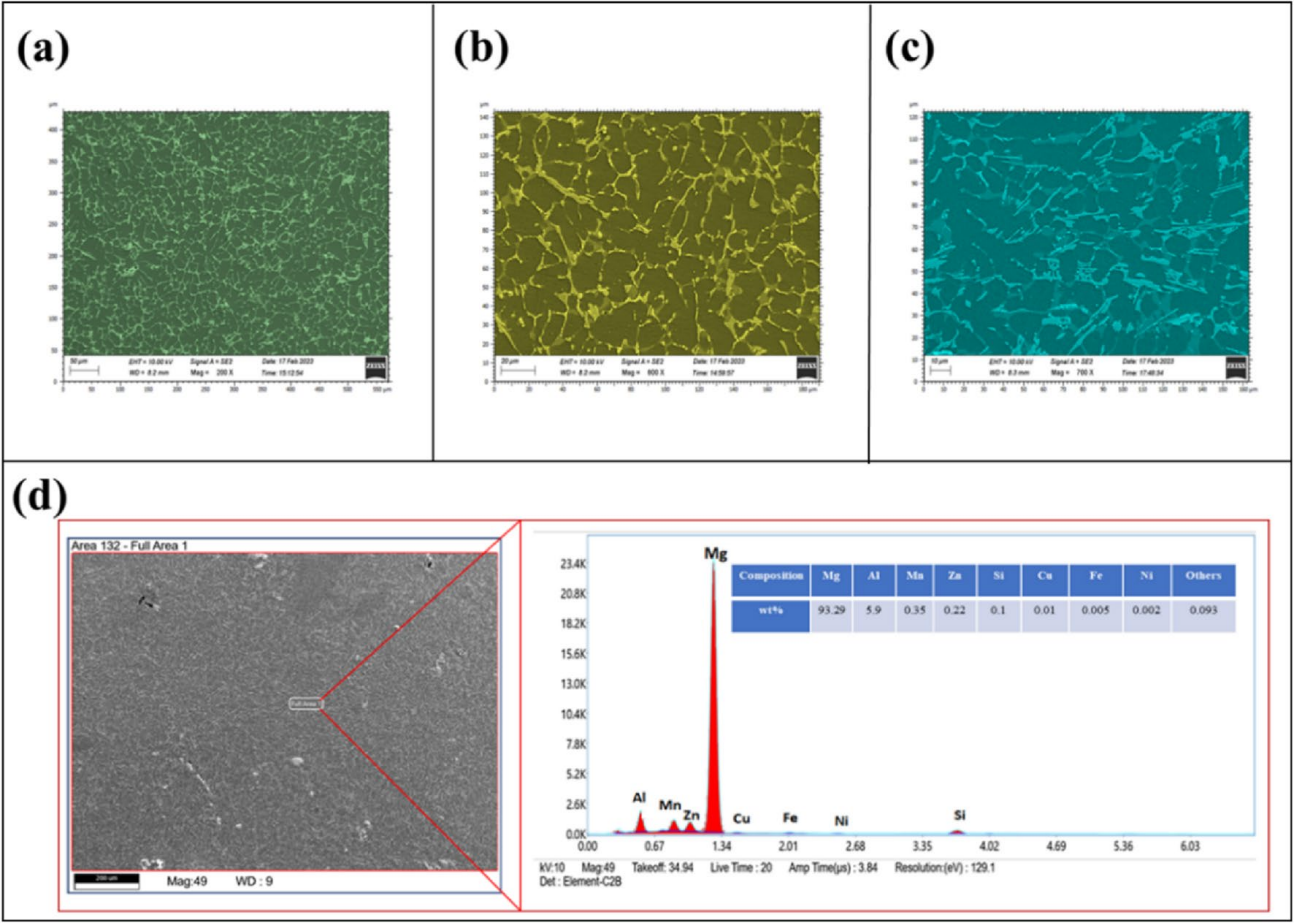
Microstructural characterization of AM60B magnesium alloy (**a**) after homogenization (**b**,**c**) before homogenization (**d**) Scanned image of AM60B at low magnification and EDS spectra measuring the composition of AM60B magnesium alloy.

The primary material utilised is commercially available AM60B as cast ingot provided by Gravity Cast Private Ltd. (located in Gujarat, India), which holds the chemical makeup that comprises the AM60B alloy as shown through Table 1 with the residual impurities. The ingot was homogenised for uniform particle distribution by heat treating in an inert atmosphere at 420 °C for 12 h. The scanning electron microscope (SEM) image of the as-cast (Fig. 1b,c) and homogenised (Fig. 1a) is shown in Fig. 1. The chemical constituents along with their percentage elements for the AM60B magnesium alloy are seen using a method called energy dispersive X-ray investigation, which is provided in Fig. 1d.

**Table 1:** Chemical composition AM60B Alloy.

Element	Mg	Al	Mn	Zn	Si	Cu	Fe	Ni
Composition	93.413	5.9	0.35	0.22	0.1	0.01	0.005	0.002

**Figure 2:**
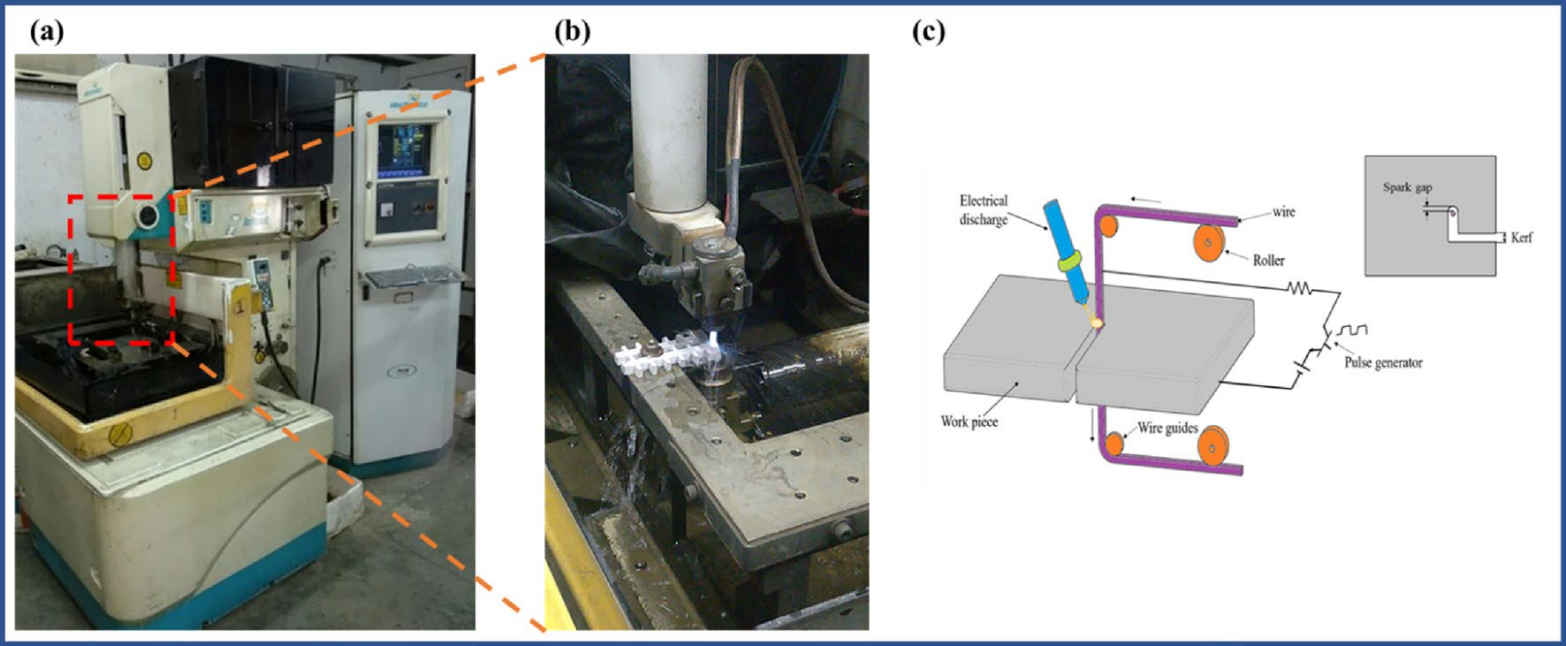
(**a**,**b**) Machining setup and (**c**) Schematic diagram of WEDM process.

The wire-cut EDM machine tool, type ELECTRONICA-SPRINTCUT, was used to complete the machining runs. Figure 2 displays an illustration of the process design. The z-direction movement limit is 300 mm, while the longitudinal and lateral travel ranges are 440 mm and 650 mm, respectively. The machine can accommodate workpieces up to an acceptable size of 700 × 800 × 300 mm. The homogenised workpiece employed in this study has dimensions of 200 × 200 × 10 mm, and the geometry for the WEDM cut that is suggested is a cube with dimensions of 10 × 10 × 10 mm. The dielectric fluid employed was deionized water, and the tool utilized was a 250 µm-diameter brass wire with a tension of 10 N and a frequency of 100 Hz.

In this research, discharge duration (D_dur_) as 105 and 125 µs, spark gap time (S_time_) as 50 and 60 µs, discharge voltage (D_volt_) as 30 and 50 V along with wire advance rate (W_adv_) as 5 and 9 mm/min are the low and high level for the factorial design. Based on early experiments, three values for each parameter are taken into consideration. Less likelihood of wire breaking during selection is indicated by the procedure parameter boundaries. Table 2 summarises the variables that can be controlled and the amount of each. Some of the fixed parameter’s values are mentioned in Table 3.

**Table 2:** Parameters of the process and the corresponding levels.

Parameters	Levels		
	1	2	3
Discharge duration (D_dur_) (µs)	105	115	125
Spark gap time (S_time_) (µs)	50	55	60
Discharge voltage (D_volt_) (V)	30	40	50
Wire advance rate (W_adv_) (mm/min)	5	7	9

**Table 3:** Fixed process parameters.

Work parameter	Description
Peak current	80 Amps
Dielectric fluid	De Ionised Water
Wire tension	10 N
Wire material	Brass

The L9 traverse array is utilised as the foundation of the experimental arrangement since Taguchi's orthogonal array will solely utilise 9 distinct arrangements of variables for obtaining equivalent data with the 81 (3^4^ experiments). In this manner, nine trials were run for surveying and arrived at the midpoint of the upsides of reactions, as in, machining rate (MR) (g/min) and surface irregularities (SR) (µm). Table 4 provides the different process parametric conditions along with the experimental outcomes. After machining each sample, weight of the physical weighing machine was used to measure all of the specimens. Calculating MR involves comparing the quantity of matter discarded to duration necessary to manufacture the specimen. The MR was determined using the Eq. (1) below.

1 $$ {\text{MR}} = \frac{{{\text{W}}_{{{\text{BM}}}} - {\text{W}}_{{{\text{AM}}}} }}{{\text{T}}} $$

where ${W}_{BM}$ and ${W}_{AM}$ are T denotes cutting time in minutes, and W represents the specimen's weight prior to and following the operation (g). A similar methodology is seen in some of the previous works (1,6). Surface irregularity (SR) is calculated employing the mathematical average roughness as a gauge (R_a_) Mitutoyo Surf test of machined surface SJ-210 series surface roughness tester. A probe is moved spanning a 10 mm expanse with a 0.8 mm threshold length, orthogonal to the path of the wire motion, in order to obtain Ra. Ra is determined three times with three separate points for every SR test, and the mean is used for tabular purposes.

**Table 4:** Experimentation results of different process parameter combinations.

Machining trails	D_dur_ (µs)	S_time_ (µs)	D_volt_ (V)	W_adv_ (mm/min)	MR (g/min)	SR (µm)
1	105	50	30	5	0.36036	4.57
2	105	55	40	7	0.47776	5.27
3	105	60	50	9	0.40504	5.35
4	115	50	40	9	0.35581	4.94
5	115	55	50	5	0.46614	5.61
6	115	60	30	7	0.49772	5.86
7	125	50	50	7	0.35516	4.61
8	125	55	30	9	0.41600	6.20
9	125	60	40	5	0.47851	5.03

**Figure 3:**
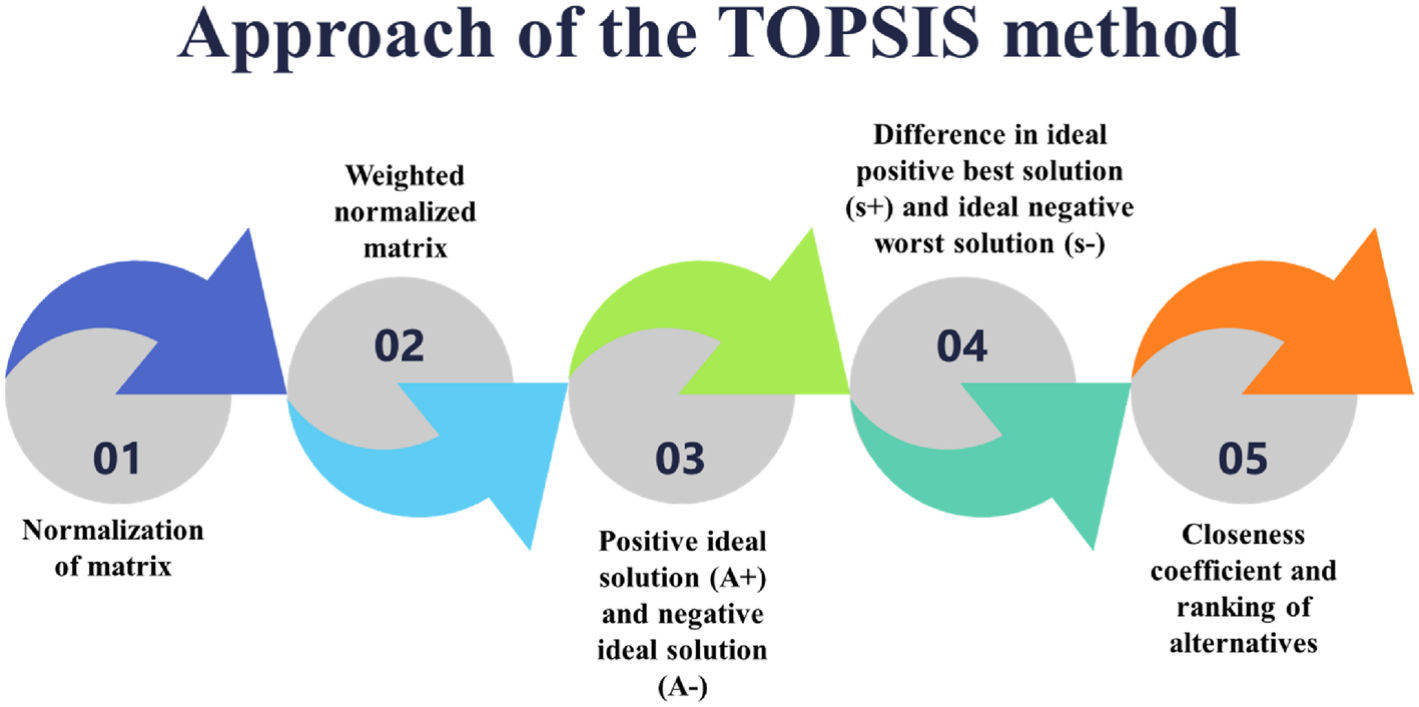
Approach of the TOPSIS method.

By requiring exact input data to resolve complex problems and assigning weights to the criteria, the TOPSIS examines the relative value of many criteria (output answers) in real-time difficulties^26^. The Critic Exam was used to apply the ranking factor. The outcome responses in this study were sorted from least to most significant, and each response was represented by a table. The decision-maker orders the value answers from least to most important based on the significance of the output responses. (e.g., machining rate, surface irregularities, micro-indentation hardness). Decision-makers use this process as a crucial tool for real-time issue analysis because it is dependable and yields findings more quickly than other weighting computational methods. The steps for proceedings are shown in Fig. 3, and a brief explanation of each step is explained below.

## Methodologies and implementations

### Desirability approach

Step 1: Determining the individual desirability index (Z_i_) with relation to the MR and SR replies. Three different types of objectives were often followed, according to the responses. Equation (2) is used since higher-is-better has been selected as the objective function.

2 $$ {\text{Z}}_{{\text{i}}} = \left\{ {\begin{array}{*{20}c} 1 & {{\hat{\text{g}}} \le {\text{g}}_{\min } } \\ {\left( {\frac{{{\hat{\text{g}}\text{j}} - {\text{g}}_{\max } }}{{{\text{g}}_{\min } - {\text{g}}_{\max } }}} \right)^{{\text{r}}} } & {{\text{g}}_{\min } \le {\hat{\text{g}}\text{j}} \le {\text{g}}_{\max } } \\ 0 & {{\hat{\text{g}}} \ge {\text{g}}_{\max } } \\ \end{array} } \right. $$

The following Eq. (3) is used when the lower-the-better target function is selected.

3 $$ {\text{Z}}_{{\text{i}}} = \left\{ {\begin{array}{ll} 1 & {{\hat{\text{g}}} \le {\text{g}}_{\min } } \\ {\left( {\frac{{{\hat{\text{g}}\text{j}} - {\text{g}}_{\min } }}{{{\text{g}}_{\min } - {\text{g}}_{\max } }}} \right)^{{\text{r}}} } & {{\text{g}}_{\min } \le {\hat{\text{g}}\text{j}} \le {\text{g}}_{\max } } \\ 0 & {{\hat{\text{g}}} \ge {\text{g}}_{\max } } \\ \end{array} } \right. $$

where g_max_ & g_min_—are the “highest and lowest value of g”.

r—weight percent value.

Step 2: Using Eq. (4), calculate the composite desirability (${\text{D}}_{\text{c}}$) value. All of the replies' individual desirability indices (Z_i_) can be added together to get a single value known as composite desirability.

4 $$ {\text{D}}_{{\text{c}}} = \sqrt {{\text{Z}}_{1}^{{{\text{w}}_{2} }} *{\text{Z}}_{2}^{{{\text{w}}_{2} }} * \cdots *{\text{Z}}_{i}^{{{\text{w}}_{{\text{i}}} }} } $$

where ${\text{z}}_{\text{i}}$ Wi is a measure of response, that represents the individual desire index. The metric for composite appeal is shown in Table 5$\left({D}_{c}\right)$ and its rank.

**Table 5:** Composite desirability (Dc) with rank.

Ex. No	Individual desirability		Composite desirability (Dc)	Rank
	MR	SR		
1	0.980	0.000	0	8
2	0.338	0.722	0.4939	5
3	0.788	0.758	0.7730	2
4	0.997	0.543	0.7360	3
5	0.435	0.862	0.6124	4
6	0.000	0.949	0	8
7	1.000	0.201	0.4482	6
8	0.736	1.000	0.8576	1
9	0.331	0.599	0.4449	7

### TOPSIS approach

#### Step 1

This TOPSIS approach begins with estimating a suitable matrix for further precedence. The accepted matrix value is calculated by using a formula, which is in Eq. (5). The accepted matrix is tabulated in Table 6.

5 $$ {\text{r}}_{{{\text{ij}}}} = \frac{{{\text{x}}_{{{\text{ij}}}} }}{{\sqrt {\sum\nolimits_{{{\text{i}} = 1}}^{{\text{m}}} {{\text{x}}_{{{\text{ij}}}} } } }} $$

*where*, ${\text{i}}$ = 1,2,3,4……0.9; ${\text{j}}$ = 1,2,3,4. ${\text{i}}$ = number of experiments. ${\text{j}}$ = number of output parameters.

**Table 6:** Accepted matrix and rounded accepted matrix.

Ex. No	Accepted matrix		Rounded accepted matrix	
	MR	SR	MR	SR
1	0.281	0.289	0.155	0.129
2	0.373	0.333	0.167	0.149
3	0.316	0.338	0.175	0.151
4	0.278	0.312	0.154	0.140
5	0.364	0.355	0.201	0.159
6	0.388	0.370	0.215	0.166
7	0.277	0.291	0.153	0.130
8	0.325	0.381	0.179	0.170
9	0.373	0.318	0.206	0.142

#### Step 2

The rounded accepted matrix $\left( {\partial_{{{\text{ij}}}} } \right)$ can be generated by multiplying the accepted matrix along with accepted value. The accepted value is calculated by using critic method. The obtained value is tabulated in Table 5. For precedence the formula is used which is in Eq. (6).

6 $$ \partial_{{{\text{ij}}}} = \left( {{\text{W}}_{{{\text{ij}}}} } \right) \times \left( {{\text{r}}_{{{\text{ij}}}} } \right) $$

*where*, ${\text{W}}_{\text{ij}}$ = Rounded Accepted Matrix. ${\text{r}}_{\text{ij}}$ = Accepted Matrix.

#### Step 3

For each rounded accepted matrix for the three output criteria. Based on the perfect great beneficial and perfect worse non-beneficial solutions are obtained. For precedence the formula is used which is in Eqs. (7) and (8).

7 $$ {\text{S}}^{ + } = \left\{ {\max \left( {\partial_{{{\text{ij}}}} } \right)} \right\}\;{\text{or}}\;\left\{ {\min \left( {\partial_{{{\text{ij}}}} } \right)} \right\} $$

8 $$ {\text{S}}^{ - } = \left\{ {\max \left( {\partial_{{{\text{ij}}}} } \right)} \right\}\;{\text{or}}\;\left\{ {\min \left( {\partial_{{{\text{ij}}}} } \right)} \right\} $$

*where,* S^+^ values for the end attributes the degree of removal and surface imperfections are [0.2147, 0.1291].

S^**−**^ values for the end attributes. The degree of removal and surface imperfections are [0.1532, 0.17013].

#### Step 4

The distances (${\text{X}}_{{\text{i}}}^{ + }$ and ${\text{X}}_{{\text{i}}}^{ - }$) between the perfect great beneficial and perfect worse non-beneficial solutions (the perfect great beneficial and perfect worse non-beneficial distances) are calculated. The following formula is used to determine its value which is in Eqs. (9) and (10).

9 $$ {\text{X}}_{{\text{i}}}^{ + } = \sqrt {\sum\nolimits_{{{\text{j}} = 1}}^{{\text{n}}} {\left( {\partial_{{{\text{ij}}}} - {\text{S}}_{{\text{j}}}^{ + } } \right)} } $$

10 $$ {\text{X}}_{{\text{i}}}^{ - } = \sqrt {\sum\nolimits_{{{\text{j}} = 1}}^{{\text{n}}} {\left( {\partial_{{{\text{ij}}}} - {\text{S}}_{{\text{j}}}^{ - } } \right)} } $$

#### Step 5

For each value, the closeness coefficient (${\text{C}}{\text{C}}_{\text{i}}$) is calculated by using a formula. The ranking is carried out based on the proximity coefficient value. A formula in Eq. (11) is utilized for precedence.

11 $$ {\text{CC}}_{{\text{i}}} = \frac{{{\text{X}}_{{\text{i}}}^{ - } }}{{{\text{X}}_{{\text{i}}}^{ + } + {\text{X}}_{{\text{i}}}^{ - } }}\;\;\;0 \le {\text{CC}}_{{\text{i}}} \le 1 $$

The fewer appealing solutions are included in Table 7, along with a closeness coefficient. The best option was picked by considering the order of the closeness coefficient (CC), which will be fairly comparable to its optimal readings. Table 7 shows a TOPSIS approach rating for various WEDM machining parameters.The performance score is displayed on the chart as being in the following numerical order: 4–9–6–7–3–2–5–8–1.

**Table 7:** Closeness coefficient with rank.

Ex. No	S +	S-	CC	Rank
1	0.059	0.041	0.409	4
2	0.052	0.025	0.326	9
3	0.046	0.029	0.386	6
4	0.062	0.031	0.329	7
5	0.032	0.049	0.603	3
6	0.036	0.062	0.628	2
7	0.062	0.040	0.393	5
8	0.054	0.026	0.327	8
9	0.015	0.060	0.796	1

**Figure 4:**
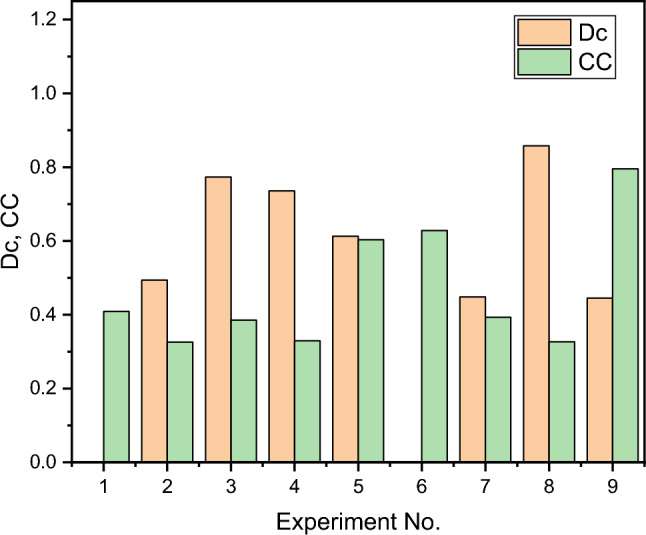
Rank plot for closeness coefficient (CC), and composite desirability (Dc).

Figure 4 depicts the sum of the ranking map for closeness coefficient (CC) with composite desirability (D_c_). The graphic reveals the test number 8 and 9 has the greatest values of CC (0.796) and Dc (0.8576), indicating that it is an ideal arrangement for the input variables is D_dur_ as 125 µs, S_time_ as 55 µs, D_volt_ 30 V, and W_adv_ as 9 mm/min, that produce manufactured composites that have decreased SR along with MR.

## Results and discussion

### Characteristics of SEM images of AM60B Magnesium Alloy

**Figure 5:**
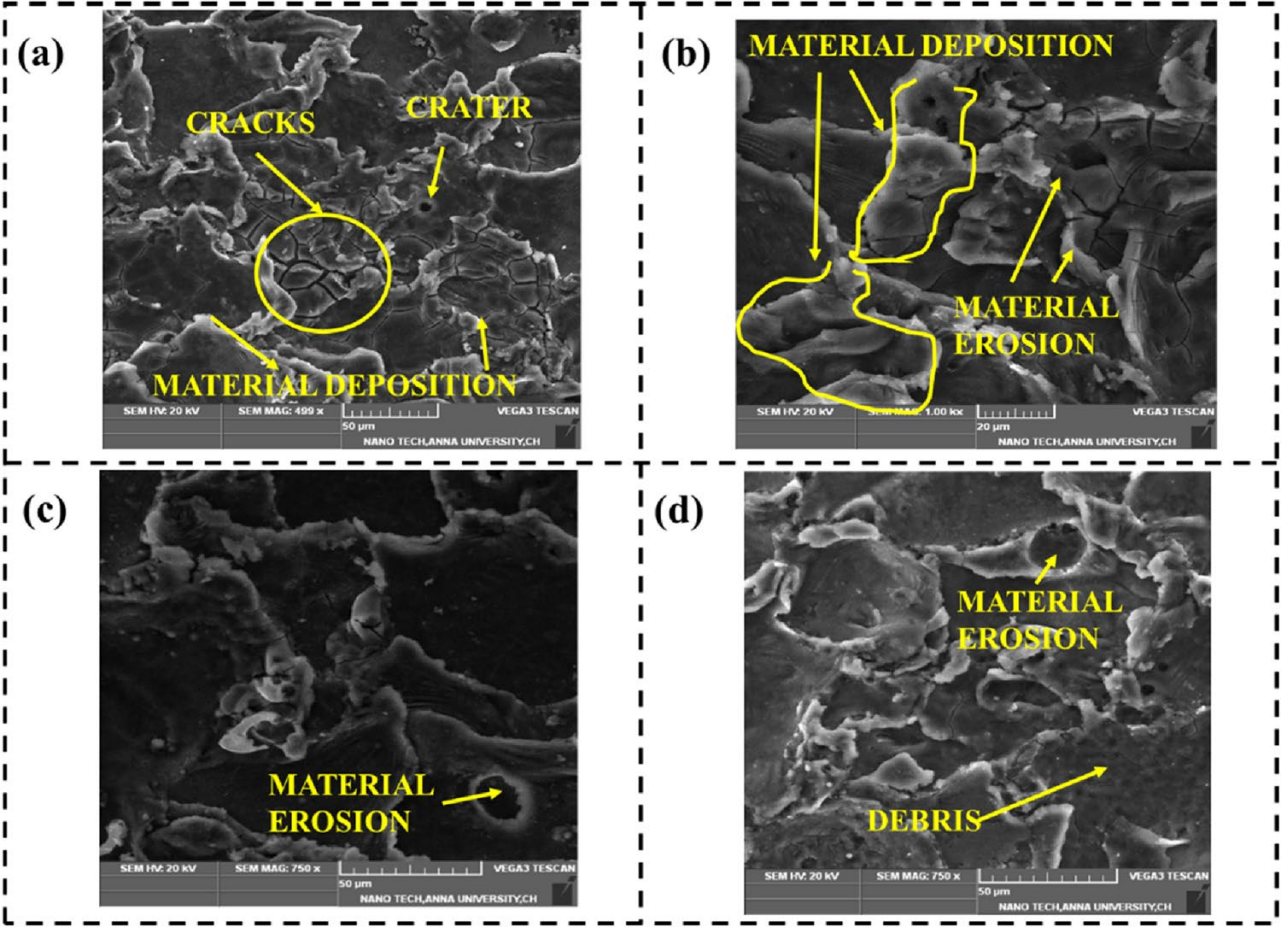
SEM image of AM60B Magnesium Alloy (**a**) higher surface irregularity (**b**) lower surface irregularity (**c**) higher machining rate (**d**) lower machining rate.

Microstructural changes and surface irregularities of the AM60B magnesium alloy were observed using a scanning electron microscope in order to investigate this effect of machining settings. Among the workpieces that were machined with various combinations of machining parameters, two were selected with higher and lower surface irregularities, as shown in Fig. 5a,b. Upon comparing the workpiece, it is evident that the recast layer thickness due to thermal distortion is higher at higher discharge durations, and the lower layer is formed at lower discharge durations. During the WEDM process, when the discharge duration is longer, more heat is generated at the machining zone, which results in the melting of material in an uncontrolled manner, causing material deposition over the surface and resulting in higher surface irregularities. The material deposition increases with the discharge duration and discharge voltage, as it causes a significant thermal burden in the space between electrodes. Another distinct feature that can be seen from the SEM images of Fig. 5a,b are craters. The microstructure reveals that utilising a higher discharge voltage causes the size of craters on the completed surface to increase. The AM60B magnesium alloy was utilised in the SEM analysis with the aim of assessing surface irregularity and characterising surface roughness levels spanning from high to low ranges of machining parameters^27^.

Similarly, Fig. 5c,d show the SEM images of the AM60B magnesium alloy with higher and lower values of machining rates. Figure 5c, depicting higher MR, is likely to show a rougher surface texture. This roughness is due to more aggressive material removal, resulting in irregularities, craters, and recast layers towards the surface^28^. Owing of their increased energy and thermal effects associated with higher MR, the presence of debris, microcracks, and erosion marks can be seen on the surface. Resolidified zones on the surface appear smaller and less pronounced due to the rapid machining process. The specific observations made on the SEM images with higher MR vary with those with lower MR, as seen in Fig. 5d. SEM images of AM60B magnesium alloy with a lower MRR are likely to exhibit a smoother and more uniform surface finish. Similarly, resolidified zones appear more extensive and well defined and show fewer signs of surface damage, such as cracks and erosion marks. The ability to yield and other mechanical characteristics associated with the alloy were significantly enhanced, which is due to the combination of strain hardening, grain refinement, and deformation. The creation of many fractures and faults in the distorted microstructure, however, caused the alloy's elongation to drop. Additionally, after dynamic deformation, many macroscopic fissures may be seen on the specimens' surfaces. As a result, while the AM60B magnesium alloy sees an increase in yield strength because of hypervelocity impact, it simultaneously experiences a reduction in ductility as a result of the appearance of fractures and other flaws^29^.

### Effects of MR on machining parameters

**Figure 6:**
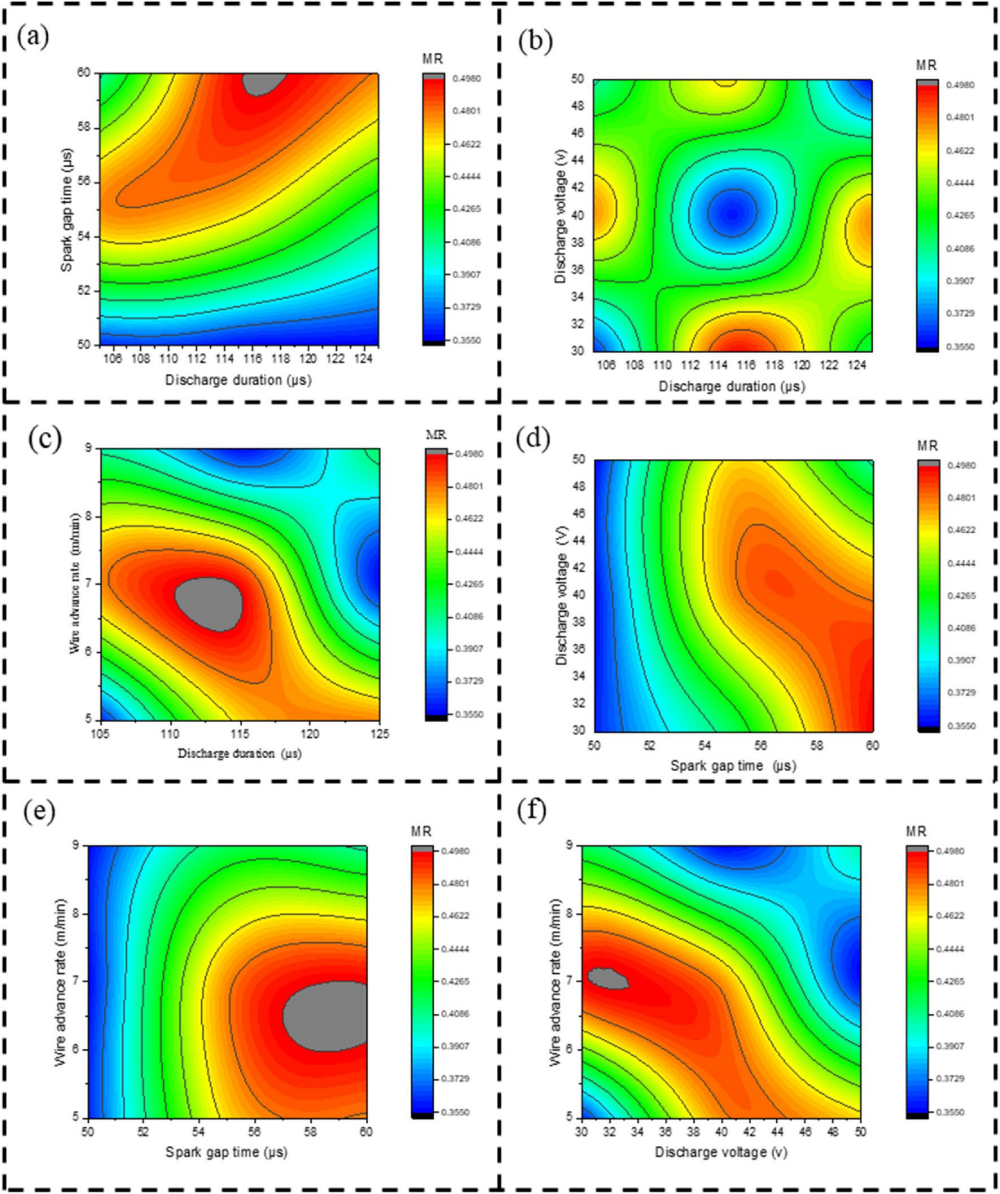
Contour plot for MR on, (**a**) D_dur_ vs S_time_ (**b**) D_dur_ vs D_volt_ (**c**) D_dur_ vs W_adv_ (**d**) S_time_ vs D_volt_ (**e**) S_time_ vs W_adv_ (**f**) D_volt_ vs W_adv_.

Figure 6a is a contour plot obtained by observing the MR for discharge duration versus spark gap time. In the graph, discharge duration is along the axis of X as well as spark gap time is along the axis of Y, where the shaded region represents MR. Where the red colour shows a high material removal rate, the green colour shows a medium material removal rate, and the dark blue colour indicates a lower material removal rate. From Fig. 6a, by increasing discharge duration at a constant low spark gap time, there is a lower MR. By increasing discharge duration at a constant high Spark gap time, at low discharge duration as medium MR, and at medium discharge duration as high MR^30^. Figure 6b is a contour plot obtained by observing the MR for discharge duration versus discharge voltage. In the graph, discharge duration is along the axis of X as well as discharge voltage is along the axis of Y, where the shaded region represents MR. From Fig. 6b, at low, medium, and high discharge durations and discharge voltages, there is less material removal rate. Other than this area, the MR seems to be high. Figure 6c is a contour plot obtained by observing the MR for discharge duration versus wire advance rate. In the graph, discharge duration is along the axis of X, and wire advance rate is along the axis of Y, in the area that is shaded represents MR. From the graph at medium to high discharge duration and wire advance rate as less MR, and less discharge duration and wire advance rate as less MR, other than the dark blue region, as more MR. Figure 6d is a contour plot obtained by observing the MR for spark gap time versus discharge voltage. In the graph, spark gap time is along the axis of X and discharge voltage is along the axis of Y, in the area that is shaded represents MR. From Fig. 6d, by increasing a discharge voltage from low to high and at a constant low spark gap time, there is less MR. At a high spark gap time for low to medium discharge voltage, the MR is high. Figure 6e is a contour plot obtained by observing the MR for spark gap time versus wire advance rate. In the graph, spark gap time is along the X axis and wire advance rate along the Y axis, where the shaded region represents MR. From Fig. 6e, by increasing a wire advance rate from low to high and at a constant low spark gap time, there is a lower MR. At a high spark gap time, the low- to medium-wire advance rate is as high as MR. Figure 6f is a contour plot obtained by observing the MR for discharge voltage versus wire advance rate. In the graph, discharge voltage is along the X axis and wire advance rate along the Y axis, where the shaded region represents MR. From the graph, from medium to high discharge voltage and wire advance rate as less MR, and less discharge voltage and wire advance rate as less MR, other than the dark blue region, as more MR.

**Figure 7:**
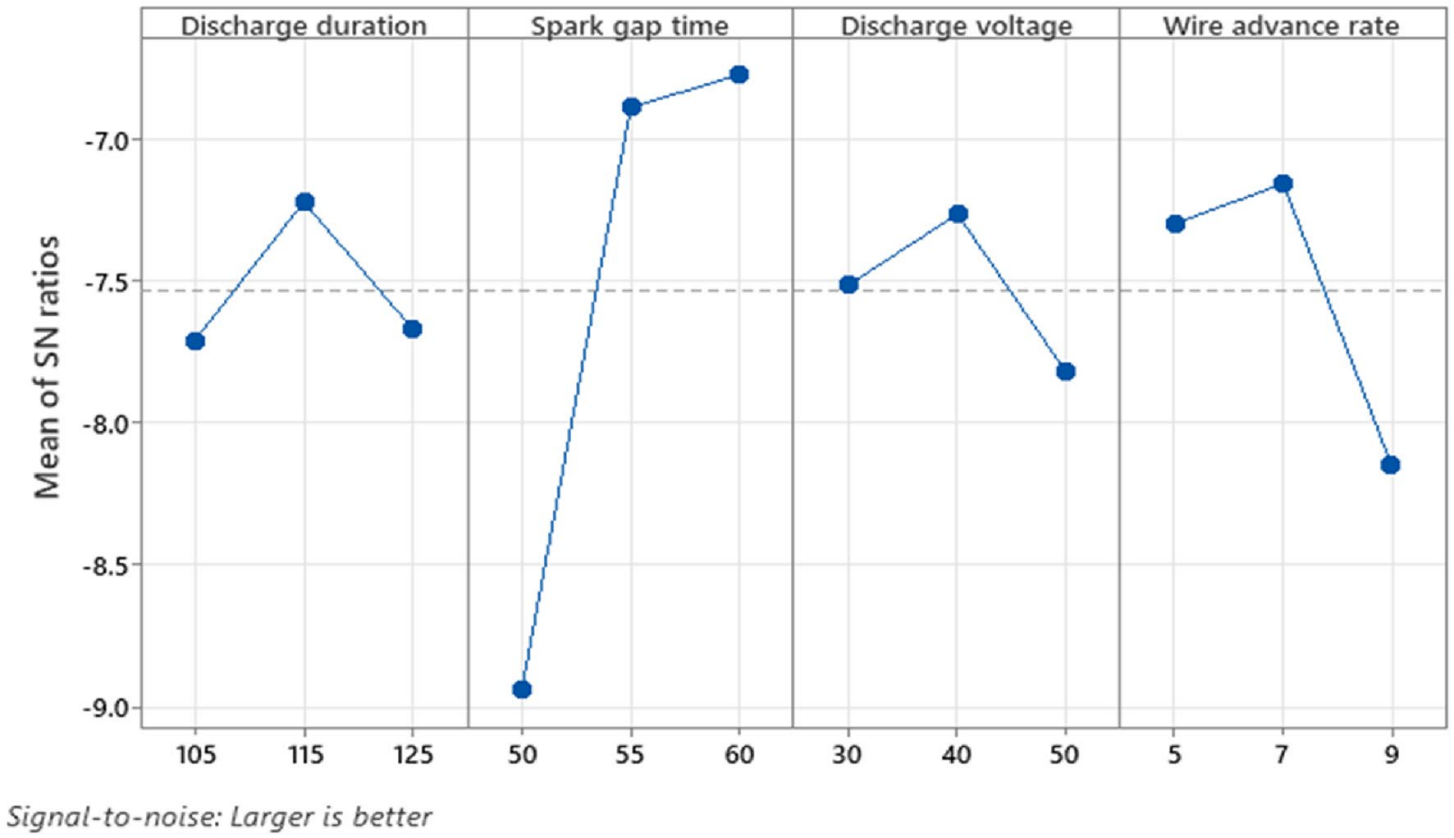
SN plot for MR.

Figure 7 is an illustration depicting the S/N ratio for MR. The graph depicts how various machining settings affect MR. This is apparent as MR decreases with lower levels for D_dur_ (125 s), S_time_ (50 s), D_volt_ (50 V), and W_adv_ (9 mm/min), respectively. The Taguchi qualitative tool prioritises the signal-to-noise (S/N) ratio for which "larger is better" the machining rate (MR). The expression 12 is used to compute the S/N ratio when greater is better. Figure 7 depicts the MR based on the S/N Ratio value. Table 8 shows the response table of the machining rate S/N ratio. S_time_ is rated first in the response table and exhibits a strong influence in MR with a difference of highest and lowest value of 2.173 dB. W_adv_ is classified as number two and plays the next important part in the MR with a difference of highest and lowest value of 0.992 dB. D_volt_ is rated third and plays the next important part in the MR with a difference of highest and lowest value of 0.559 dB. D_dur_ has a little impact on MR, with a difference of highest and lowest value of 0.489 dB, and is rated at position 4. The ANOVA findings for MR is displayed by Table 9. According to data presented in table, prominent factor, S_time_, has an F-value of 8.21, subsequent to W_adv_, and possesses a the F-value around 1.4, and D_volt_, and possesses a the F-value around 0.19. Additionally, D_dur_ has an F-value of 0.00, making it a less important factor than the rest. The main prominent components are S_time_, W_adv_, and D_volt_, with contributions of 59.44%, 10.16%, and 1.41%%, respectively. D_dur_ has a contribution of 0.025%, which makes it a less important element. The R^2^ and R^2^(adj) values of 71.05% and 42.09% respectively show demonstrating the model is capable of accurately predicting its MR provided with an input variable. In this instance, as S_time_ rises, the MR rises gradually. Due to the increased D_volt_ in the machining zone caused by the maximum MR produced at a higher level of S_time_, the work piece's surface is therefore marred by craters and voids. Additionally, during machining, the integration of reinforcement particles does not melt, leading to the creation of a rough surface. Similar to this, the longer D_dur_ generates more MR since the ember that exists within the cutting instrument and the work piece continues longer.

12 $$ {\text{S}}/{\text{N}}\;{\text{ratio}} = - 10\log \left( {\frac{{1/\sum {\text{y}}^{2} }}{{\text{n}}}} \right) $$

**Table 8:** Response table for S/N ratios for MR.

Level	D_dur_	S_time_	D_volt_	W_adv_
	(µs)	(µs)	(V)	(mm/min)
1	7.71	8.944	7.515	7.299
2	7.222	6.888	7.265	7.156
3	7.671	6.771	7.824	8.148
Delta	0.489	2.173	0.559	0.992
Rank	4	1	3	2

**Table 9:** ANOVA table for machining rate.

Source	*df*	*Adj SS*	*Adj MS*	*f-Value*	*p-Value*
Model	4	0.019135	0.004784	2.45	0.203
Discharge duration	1	0.000007	0.000007	0	0.955
Spark gap time	1	0.01601	0.01601	8.21	0.046
Discharge voltage	1	0.00038	0.00038	0.19	0.682
Wire advance rate	1	0.002737	0.002737	1.4	0.302
Error	4	0.007798	0.00195		
Total	8	0.026933			

Source	R-sq	R-sq(adj)	R-sq(pred)		
0.0441532	71.05%	42.09%	0.00%		

### Effects of SR on machining parameters

**Figure 8:**
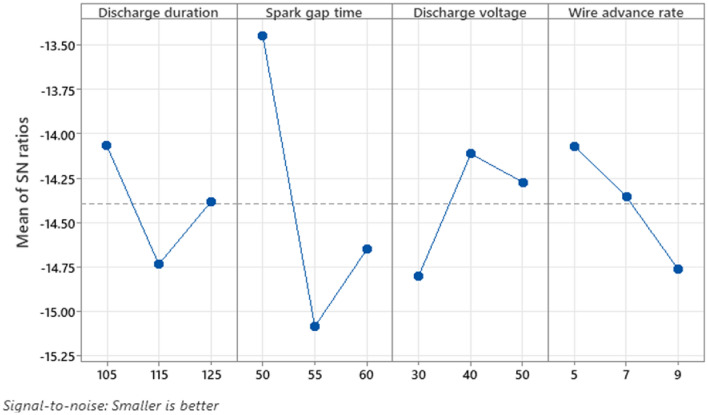
SN plot for SR.

Figure 8 depicts the graph of the S/N ratio for SR. The illustration shows how machining settings affect SR. It is apparent as SR decreases at low levels of D_dur_ (105 s), Stime (50 s), D_volt_ (40 V), and W_adv_ (five metres per minute, respectively). The Taguchi qualitative tool's "smaller the better" S/N ratio for the SR is favoured. The expression 13 is used to compute the S/N ratio when smaller is better. Figure 8 depicts the SR based on the S/N Ratio value. Table 10 shows the response table for the surface roughness S/N ratio. S_time_ is positioned as the primary variable in the response table and has a significant association with SR, as shown by a difference of highest and lowest value of 1.64 dB. W_adv_ is categorised as the second-ranked factor and has a significant impact on the SR, as seen by its difference of highest and lowest value of 0.69 dB. D_volt_, ranked third, fulfils a subsequent important part in the SR and has a value difference between the highest and lowest 0.69 dB. D_dur_ is placed fourth and has the smallest role in SR with a difference of highest and lowest value of 0.67 dB. The findings of the ANOVA for SR are shown in Table 11. This is clear through the table with the most significant component, S_time_, has an F-value of 2.61, followed by W_adv_ (0.95), then D_volt_ (0.65), and then W_adv_ again (0.95). D_dur_ also has an F-value of 0.25, making it a non-significant factor in comparison to the others. The three main significant components are S_time_, W_adv_, and D_volt_, each contributing 30.86%, 11.25%, and 7.71%%, respectively. D_dur_ has a contribution of 2.90%, making it a less important element. R^2^ and R^2^(adj) values of 52.73% and 5.46% respectively show that the model is effective in predicting the SR for an input parameter. In this instance, as S_time_ grows, the SR rises gradually. So that craters and voids are produced as an outcome from the maximal SR obtained at a greater magnitude of S_time_ due to higher D_volt_ in the zone of machining on the job piece's surface.

13 $$ {\text{S}}/{\text{N}}\;{\text{ratio}} = - 10\log \left( {\frac{{\sum {\text{y}}^{2} }}{{\text{n}}}} \right) $$

**Table 10:** S/N ratio for surface irregularity.

Level	D_dur_	S_time_	D_volt_	W_adv_
	(µs)	(µs)	(V)	(mm/min)
1	− 14.07	− 13.45	− 14.8	− 14.07
2	− 14.74	− 15.09	− 14.11	− 14.36
3	− 14.38	− 14.65	− 14.27	− 14.76
Delta	0.67	1.64	0.69	0.69
Rank	4	1	3	2

**Table 11:** ANOVA for surface irregularity.

Source	*df*	*Adj SS*	*Adj MS*	*f-Value*	*p-Value*
Model	4	1.27982	0.31995	1.12	0.459
Discharge duration	1	0.07042	0.07042	0.25	0.646
Spark gap time	1	0.74907	0.74907	2.61	0.181
Discharge voltage	1	0.18727	0.18727	0.65	0.464
Wire advance rate	1	0.27307	0.27307	0.95	0.384
Error	4	1.14727	0.28682		
Total	8	2.42709			

Source	R-sq	R-sq(adj)	R-sq(pred)		
0.535554	52.73%	5.46%	0.00%		

**Figure 9:**
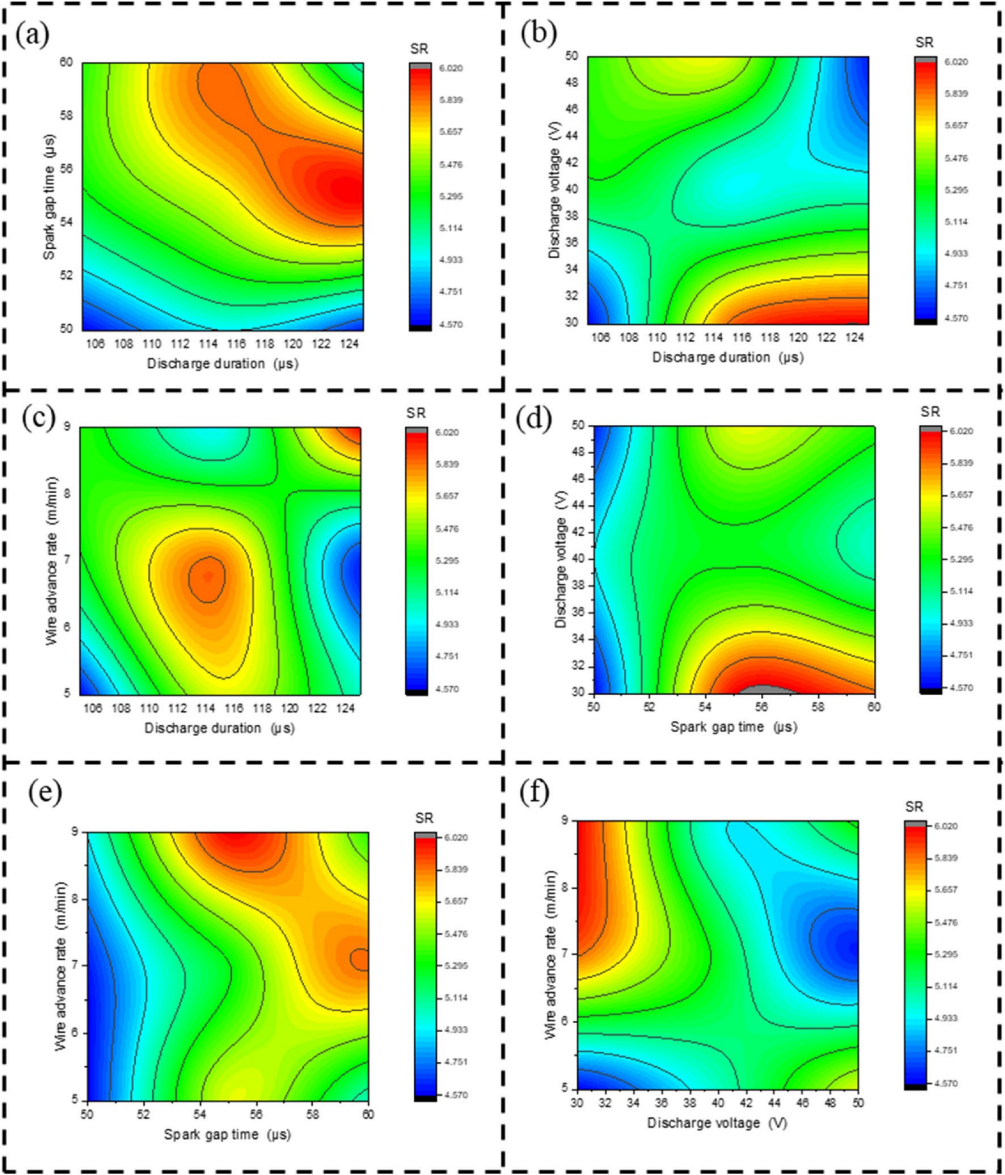
Contour plot for surface irregularity on, (**a**) D_dur_ vs S_time_ (**b**) D_dur_ vs D_volt_ (**c**) D_dur_ vs W_adv_ (**d**) S_time_ vs D_volt_ (**e**) S_time_ vs W_adv_ (**f**) D_volt_ vs W_adv_.

Figure 9a is contour plot by observing the SR for discharge duration verses spark gap time. In the graph, discharge duration is along the axis of X and spark gap time is along the axis of Y, where the shaded region represents SR. Where the red colour shows high surface irregularities, the green colour shows medium surface irregularities, and the dark blue colour indicates less surface irregularities. From Fig. 9a, increasing discharge duration at a constant low spark gap time has a lower SR. high discharge duration at a constant high Spark gap time, at low discharge duration as medium SR, and at medium discharge duration as high SR^27^. Figure 9b is a contour plot obtained by observing the SR for discharge duration versus discharge voltage. In the graph, discharge duration is along the axis of X and discharge voltage is along the axis of Y, where the shaded region represents SR. From Fig. 9b, at low, medium, and high discharge duration and discharge voltage, there are fewer surface irregularities. Other than this area, the SR seems to be high. Figure 9c is a contour plot obtained by observing the SR for discharge duration versus wire advance rate. In the graph, discharge duration is along the X axis and wire advance rate along the Y axis, where the shaded region represents SR. From the graph, at high discharge duration and at low to medium wire advance rate, there is less SR, and at low discharge duration and wire advance rate, there is less SR. other than the dark blue region as more SR. Figure 9d is a contour plot obtained by observing the SR for spark gap time versus discharge voltage. In the graph, spark gap time is along the axis of X and discharge voltage is along the axis of Y, where the shaded region represents SR. From Fig. 9d, by increasing a discharge voltage from low to high and at a constant low spark gap time, there is less SR. At medium to high spark gap time for low to medium discharge voltage as high SR. Figure 9e is a contour plot obtained by observing the SR for spark gap time versus wire advance rate. In the graph, spark gap time is along the X axis and wire advance rate along the Y axis, where the shaded region represents SR. From Fig. 9e, by increasing a wire advance rate from low to high and at a constant low spark gap time, there is a lower SR. At medium to high spark gap time, the low to medium wire advance rate is as high as SR. Figure 9f is a contour plot obtained by observing the SR for discharge voltage versus wire advance rate. In the graph, discharge voltage is along the X axis and wire advance rate along the Y axis, where the shaded region represents SR. From the graph, medium to high discharge voltage and medium to high wire advance rate are less SR, and less discharge voltage and less wire advance rate are less SR. other than the dark blue region as more SR.

### Effects of closeness coefficient on machining parameters

**Figure 10:**
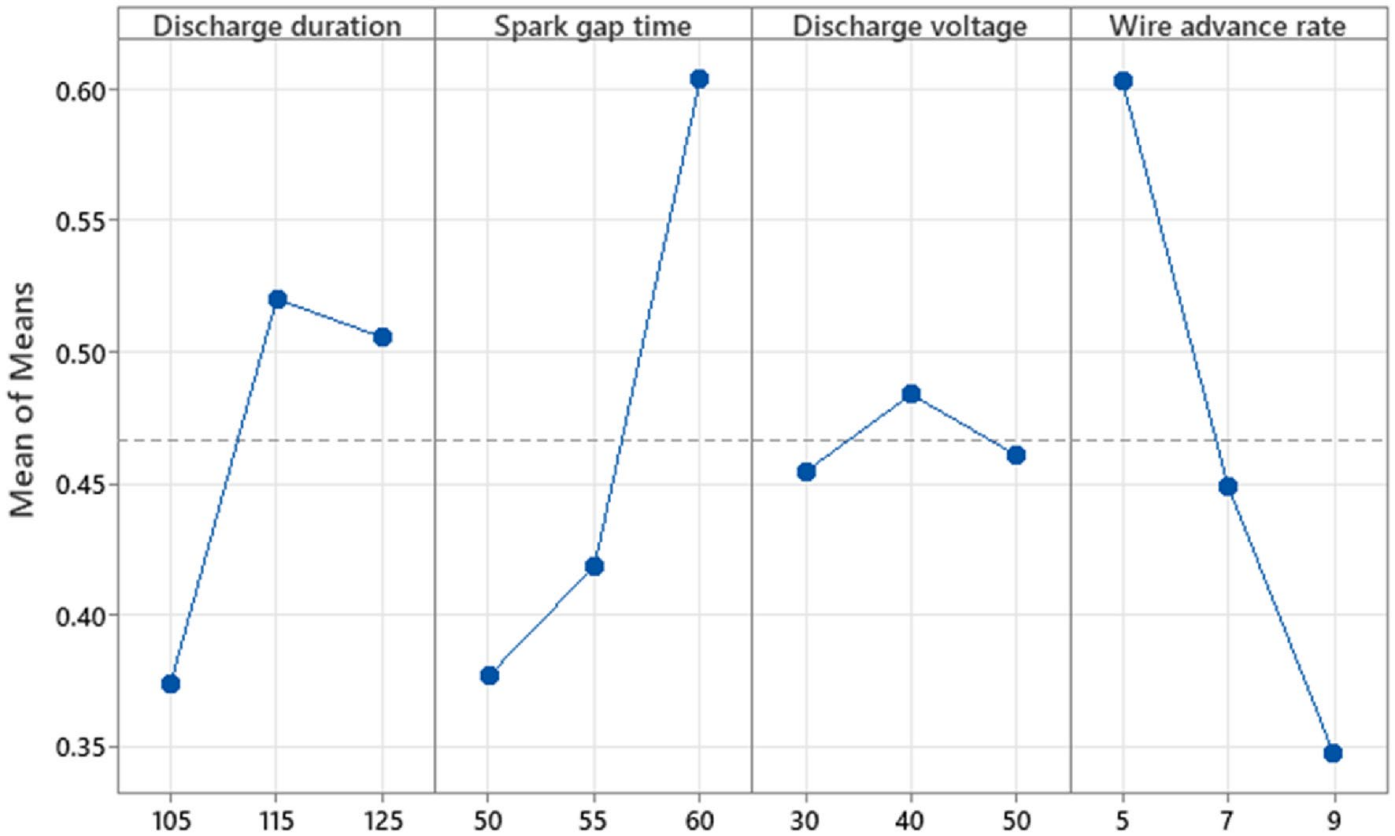
Mean plot for closeness coefficient.

**Figure 11:**
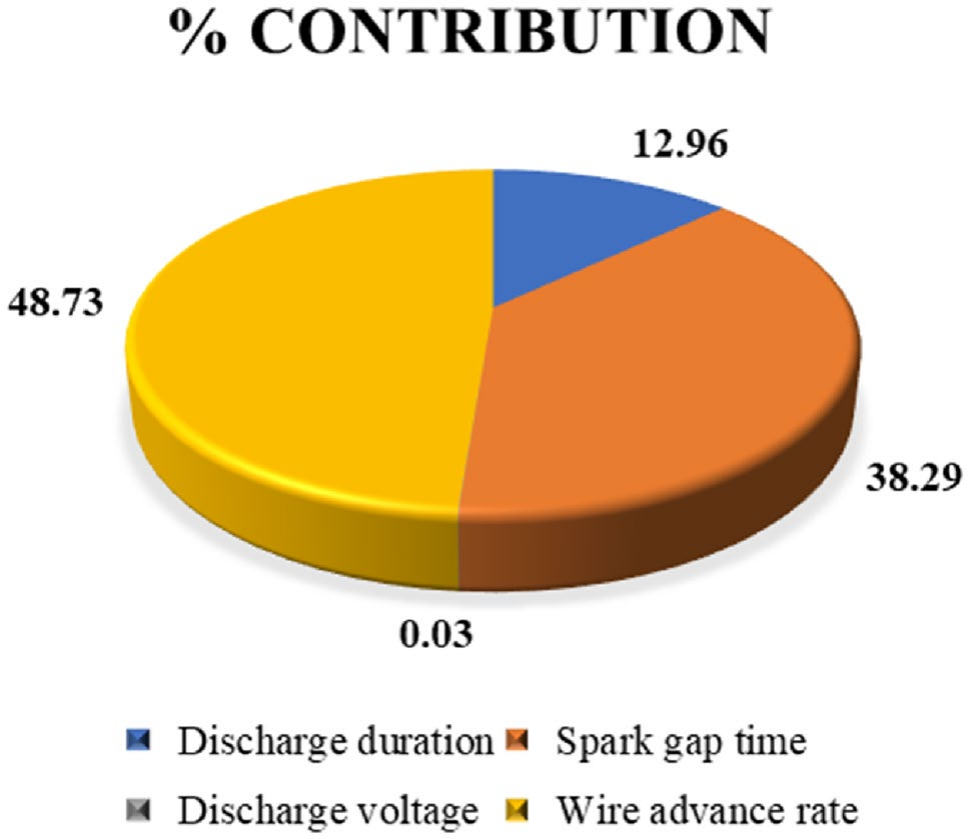
Percentage contribution of parameters.

The mean plot for closeness coefficient alongside the various parameters used to machine is depicted visually in Fig. 10. The graph depicts the ideal settings for achieving a greater machining rate with lesser surface irregularities for AM60B magnesium alloy. It is evident from the figure that the best machining settings comprise discharge time during level 2 (115 µs), spark gap time at level 3 (60 µs), discharge voltage at wire advancement frequency at level 1 and level 2 (40 V) (5 mm/min). It can be seen that the wire advance rate is another significant element in MR and SR. When the wire advance rate is greater, the amount of discharge duration and the low level of discharge voltage produce more MR^31^. This is due to the fact that when discharge duration is turned on for a longer period, more particles are included in the workpiece, which results in a higher MR^32^. Hence, longer discharge duration increases MR and increases SR as well^33^. The increase in surface irregularity this is because to the heat generated in the machining zone and the longer discharge duration made the spark gaps less stable, leading to variation in the discharge process. Table 12 shows an outcome using ANOVA with a closeness coefficient. From Table 10 it is evident that F-value of W_adv_ is 15.11 and S_time_ is 11.87, is more than that of D_dur_ (4.02). Hence it is clear that these two factors are considered to be more significant and influences MR and SR during WEDM process of AM60B magnesium alloy. Table 13 shows the mean table for closeness coefficient. From response table, the delta value of W_adv_ is 0.2553 which is ranked 1, S_time_ is 0.2263 ranked 2, D_dur_ is 0.1163 ranked 3 and D_volt_ is 0.029 ranked 4. The discharge voltage does not have any influence in WEDM process of AM60B magnesium alloy. Figure 11 provides the percentage combination of parameters influencing WEDM of AM60B alloy. Wire advance rate is a noteworthy factor which contributes 43.16%, next by spark gap time with 33.91%, while discharge duration is 11.48%.

**Table 12:** Analysis of variance for closeness coefficient.

Source	*df*	*Adj SS*	*Adj MS*	*f-Value*	*p-Value*
Model	4	0.200691	0.050173	7.75	0.036
Discharge duration	1	0.026004	0.026004	4.02	0.116
Spark gap time	1	0.07684	0.07684	11.87	0.026
Discharge voltage	1	0.000054	0.000054	0.01	0.932
Wire advance rate	1	0.097793	0.097793	15.11	0.018
Error	4	0.025889	0.006472		
Total	8	0.22658			

Source	R-sq	R-sq(adj)	R-sq(pred)		
0.0804503	88.57%	77.15%	55.79%

**Table 13:** Mean table for closeness coefficient.

Level	D_dur_	S_time_	D_volt_	W_adv_
	(µs)	(µs)	(V)	(mm/min)
1	0.3737	0.377	0.4547	0.6027
2	0.52	0.4187	0.4837	0.449
3	0.5053	0.6033	0.4607	0.3473
Delta	0.1463	0.2263	0.029	0.2553
Rank	3	2	4	1

### Confirmation test

Table 14 presents a comparative analysis of two distinct methodologies, namely the approach for Desirability and the Technique for Order of Preference by Similarity to Ideal Solution (TOPSIS) Approach. This table aims to optimise a collection of parameters within an industrial or experimental setting. Various methodologies are employed to assess the effects of distinct combinations of input parameters (A, B, C, and D) on specified output parameters (MR and SR), taking into account a predefined set of goal values. The Desirability technique yields the most improvement at 4.56% and 4.193% for the MR and SR, respectively. The TOPSIS technique yields the highest level of improvement at 1.77% and 2.78% for the MR and SR, respectively.

**Table 14:** The % improvement of machining parameters.

Parameters	D_dur_	S_time_	D_volt_	W_adv_	MR	SR	% improvement	
Units	(µs)	(µs)	(V)	(mm/min)	(g/min)	(µm)	MR	SR
Desirability approach								
A3B2C1D3	125	55	30	9	0.416	6.2	4.56	4.193
A3B2C1D3	125	55	30	9	0.435	5.94		
TOPSIS Approach								
A3B3C2D1	125	60	40	5	0.47851	5.03	1.77	2.78
A2B3C2D1	115	60	40	5	0.487	4.89		

## Conclusion

In this work, the AM60B magnesium alloy has been machined using WEDM, and the machinability behaviour has een analyzed. The ideal parameter constraint in WEDM, including discharge duration (D_dur_), spark gap time (S_time_), discharge voltage (D_volt_) and wire advance rate (W_adv_) for attaining maximum machining rate (MR) and minimum surface irregularity (SR), was studied utilising the Taguchi combined TOPSIS and desirability method.

- According to Taguchi findings, the machining rate rises at a given set up D_dur_ (115 µs), S_time_ (60 µs), D_volt_ (40 V) and W_adv_ (7 mm/min). Similarly surface irregularity decreases at parameter combination of D_dur_ (115 µs), S_time_ (55 µs), D_volt_ (30 V) and W_adv_ (9 mm/min).
- In accordance with TOPSIS data, experiment 9 has the greatest composite desirability score of 0.8576, and the associated levels of parameters for machining are more in line with ideal circumstances.
- From closeness coefficient graph, it is revealed that the maximum machining rate (MR) and the minimum surface irregularity (SR) is attained at 115 µs of discharge duration, 60 µs of spark gap time, 40 V of discharge voltage and 5 mm/min of wire advance rate.
- ANOVA results confirmed the order of significant parameter affecting MR and SR and wire advance rate having significant contribution with 43.16%, followed by spark gap time with a contribution of 33.91%.
- AM60B magnesium alloys, known for their low conductivity, make them prone to thermal effects during machining process. The spark gap time, directly influences the amount of energy applied to the workpiece and controlling the heat input becomes critical to avoid excessive thermal damage. A longer spark gap time allows for better control over the heat input, preventing the material from overheating and minimizing the risk of thermal damage. AM60B magnesium alloy is sensitive to wire advance rate variations as they are to spark gap time due to their thermal characteristics. Similarily variations in discharge voltage and duration does not have pronounced effects on heat input and thermal effects, especially when compared to the direct control provided by spark gap time.
- SEM images of higher and lower values of MR and SR indicate the presence of various microstructural changes due to the thermal burden created on the machined surface. Presence of craters, recast layers, microcracks, and the formation of fissures at various ranges of machining parameters.

## Data Availability

Since the current work is connected to a PhD thesis, the datasets created and evaluated are not publicly available. It would be delivered upon the submission of the research study. Nonetheless, on reasonable request, are available from the corresponding author.

## Abbreviations

WEDM: Wire electrical discharge machining
MR: Machining rate
SR: Surface irregularities
ANOVA: Analysis of Variance
D_dur_: Discharge duration
S_time_: Spark gap time
D_volt_: Discharge voltage
W_adv_: Wire advance rate
